# A multifunctional targeting probe with dual-mode imaging and photothermal therapy used in vivo

**DOI:** 10.1186/s12951-018-0367-9

**Published:** 2018-04-19

**Authors:** Xiao-Shuai Zhang, Yang Xuan, Xiao-Quan Yang, Kai Cheng, Ruo-Yun Zhang, Cheng Li, Fang Tan, Yuan-Cheng Cao, Xian-Lin Song, Jie An, Xiao-Lin Hou, Yuan-Di Zhao

**Affiliations:** 1 Britton Chance Center for Biomedical Photonics at Wuhan National Laboratory for Optoelectronics–Hubei Bioinformatics & Molecular Imaging Key Laboratory, Department of Biomedical Engineering, College of Life Science and Technology, Huazhong University of Science and Technology, Wuhan, 430074 Hubei People’s Republic of China; 2 Key Laboratory of Optoelectronic Chemical Materials and Devices, Ministry of Education, Jianghan University, Wuhan, 430056 People’s Republic of China; 3 Key Laboratory of Biomedical Photonics (HUST), Ministry of Education, Huazhong University of Science and Technology, Wuhan, 430074 Hubei People’s Republic of China

**Keywords:** Ag_2_S, Cancer active targeting, Fluorescence imaging, Photoacoustic imaging, Photothermal therapy

## Abstract

**Background:**

Ag_2_S has the characteristics of conventional quantum dot such as broad excitation spectrum, narrow emission spectrum, long fluorescence lifetime, strong anti-bleaching ability, and other optical properties. Moreover, since its fluorescence emission is located in the NIR-II region, has stronger penetrating ability for tissue. Ag_2_S quantum dot has strong absorption during the visible and NIR regions, it has good photothermal and photoacoustic response under certain wavelength excitation.

**Results:**

200 nm aqueous probe Ag_2_S@DSPE-PEG_2000_-FA (Ag_2_S@DP-FA) with good dispersibility and stability was prepared by coating hydrophobic Ag_2_S with the mixture of folic acid (FA) modified DSPE-PEG_2000_ (DP) and other polymers, it was found the probe had good fluorescent, photoacoustic and photothermal responses, and a low cell cytotoxicity at 50 μg/mL Ag concentration. Blood biochemical analysis, liver enzyme and tissue histopathological test showed that no significant influence was observed on blood and organs within 15 days after injection of the probe. In vivo and in vitro fluorescence and photoacoustic imaging of the probe further demonstrated that the Ag_2_S@DP-FA probe had good active targeting ability for tumor. In vivo and in vitro photothermal therapy experiments confirmed that the probe also had good ability of killing tumor by photothermal.

**Conclusions:**

Ag_2_S@DP-FA was a safe, integrated diagnosis and treatment probe with multi-mode imaging, photothermal therapy and active targeting ability, which had a great application prospect in the early diagnosis and treatment of tumor.

**Electronic supplementary material:**

The online version of this article (10.1186/s12951-018-0367-9) contains supplementary material, which is available to authorized users.

## Background

Early diagnosis of cancer can greatly improve the survival of patients [1]. More and more imaging methods have been applied to diagnose tumor, such as electronic computed tomography (CT), photoacoustic imaging (PAI), magnetic resonance imaging (MRI), positron emission computed tomography (PET), fluorescence imaging (FI) and so on.

As a molecular imaging technique, FI has the advantages of high resolution and low acquisition time, it can observe the dynamic change of tumor-related molecules in real time, so it has great application prospect in early diagnosis [2–4]. However, some endogenous substances (hemoglobin, water, bilirubin and melanin, etc.) in biological tissues have strong absorption and scattering for visible light (400–700 nm) [5], which result in the limited penetration of photon. By contrast, near-infrared (NIR) light has stronger penetrating ability because of weak tissue scattering and absorption in that region, so NIR fluorescent materials usually are used as probe for in vivo imaging [6]. Ag_2_S is a new type of low-toxic quantum dot reported in recent years. It has the characteristics of conventional quantum dot such as broad excitation spectrum, narrow emission spectrum, long fluorescence lifetime, strong anti-bleaching ability, and other optical properties [7]. Moreover, since its fluorescence emission is located in the NIR-II region, it has stronger penetrating ability for tissue and can also eliminate the interference of biological spontaneous fluorescence, which contributes to the improvement of signal-to-noise ratio [8]. So it is a promising quantum dot for in vivo imaging.

PAI is a new kind of molecular imaging technology developed in recent years [9]. In PAI, a short pulsed laser beam is used to illuminate the biological tissue. The absorption of laser pulse energy of the tissue then induces an instantaneous temperature rise and transient thermoelastic expansion, leading to ultrasonic emissions (photoacoustic waves). Researchers can map the light absorption distribution in the tissues by measuring the photoacoustic (PA) signals [10]. PAI combines the advantages of high-contrast of optical imaging and high penetration depth of ultrasound imaging, avoids the effect of light scattering in principle, can achieve the deep tissue imaging similar to ultrasound imaging and high-contrast imaging similar to optical coherence tomography, demonstrates broad prospect for early diagnosis and efficacy monitoring [11, 12]. Ag_2_S quantum dot has strong absorption during the visible and NIR regions [13], it has good photoacoustic response under certain wavelength excitation, so it is also a good PAI contrast agent.

Single imaging model often cannot provide comprehensive information due to the existence of certain defects. Combining two or more imaging technologies is an inevitable trend in the development of in vivo molecular imaging technology, which is also an effective way to early diagnose and treat cancer. So multi-modal imaging technology comes into being [14–16]. FI can give the three-dimensional quantitative results of fluorescence molecules in vivo, but there are some shortcomings in obtaining structural information; PAI can provide high-resolution and high-contrast tissue imaging. Therefore, combining FI and PAI is an ideal dual-mode molecular imaging method [17]. FI provides the distribution and location of trace cancer-related molecules in tissues, while PAI provides deep tissue and spatial structure information.

Thermotherapy as an effective treatment for cancer has aroused widespread attention [18–21]. Tumor thermotherapy uses the physical energy to heat, make the tumor temperature rise to the effective treatment temperature (> 41 °C) [22] and maintain a certain time. Due to different temperature tolerance comparing with normal cell, tumor cell apoptosis is achieved without damaging the normal cell. Photothermal therapy (PTT) research in the past is mainly using NIR organic dyes [23]. In recent years, studies have shown that absorption cross sections of some metal nanoparticles are 4–5 times larger than the conventional dyes [24], and have better stability and stronger anti-photobleaching, so they become the ideal choice for PTT. It is gratifying that, Ag_2_S quantum dot had significant photothermal effect reported by Wu et al. in 2016 [25]. It is thought that Ag_2_S quantum dot can achieve NIR fluorescence and photoacoustic dual-mode imaging, meanwhile be used to kill the tumor through photothermal [26], it achieves the purpose of diagnosis and treatment integration perfectly.

In this paper, a multi-functional folic acid (FA) modified phospholipid coating Ag_2_S probe (Ag_2_S@DSPE-PEG_2000_-FA) with low biotoxicity was prepared by mixing lecithin, polyoxyethylene stearate, DSPE-PEG_2000_ (DP) and FA modified DP (DP-FA). The design idea is that DP is a very safe amphiphilic molecule whose hydrophilic end can improve the water solubility of the nanoprobe and help the probe to escape the phagocytosis of the reticuloendothelial system and prolong the cycle time in the body [27]. However, due to its limited emulsifying ability, lecithin and polyoxyethylene stearate are required to assist. These two materials can be effectively utilized by the body and have little hemolysis effect, are safe and reliable injection emulsion surfactant. FA, as a small molecule of vitamin, can bind FA receptors which are highly expressed on the surface of some tumor cells to guide the endocytosis, while it is easily coupled with nanoparticles, free of immunogenicity and low cost. Two kinds of cells were adopted, while HeLa cells (high FA receptor expression) [28] as positive cell and A549 cells (low FA receptor expression) [29] as negative cell; two kinds of probes were also adopted, while Ag_2_S@DP-FA as positive probe and Ag_2_S@DP as negative probe. Cross-contrast experiments were performed by fluorescence, photoacoustic and photothermal, the results confirmed that Ag_2_S@DP-FA probe was an integrated diagnosis and treatment probe with good safety, it had abilities of fluorescence and photoacoustic dual-mode imaging, PTT and active targeting function, had large application prospect in the early diagnosis and treatment of tumor.

## Methods

### Materials and instrument

Diethyldithiocarbamic acid silver salt (AgDDTC, 98%), dodecanethiol (DT, 98%), *n*-hexane, acetone, trichloromethane, dimethyl sulfoxide (DMSO), folic acid (FA, 97%) and pyridine were purchased from Sinopharm Group Chemical Reagent Co., Ltd. PEG-phospholipid (DSPE-PEG_2000_), amino-PEG-phospholipid (DSPE-PEG_2000_-NH_2_) were purchased from Avanti, lecithin (98%), polyoxyethylene stearate were purchased from Aladdin, octadecene (ODE, 90%) was purchased from Aldrich. All reagents were used directly without further purification.

The equipments used in the experiments included UV-2550 UV–Vis spectrophotometer (Shimadzu, Japan), Tecnai G20 U-Twin High Resolution Transmission Electron Microscope (FEI, Netherlands), Nano-ZS90 Nanometer Size Meter (Malvern, UK), Ni-E Positive Slice Scanning Microscope (Nikon, Japan), SP-4430 Dry Biochemical Analyzer (Arkary, Japan), Centrifugal Concentrator (Ependorf, Germany), Elx-808 Microplate Reader (Biotek, USA), CA-700 Automatic Blood Analyzer (STAC, China), MDL-III-808-2.5 W Laser (Changchun New Industries Optoelectronics Tech. CO., LTD., China), EasIR-9 Thermal Imager (Wuhan Guide Infrared Co., LTD, China), IX71 Fluorescence Microscope (Olympus, Japan). Near infrared FI system [30] and PAI system [31, 32] were homemade by the laboratory.

### Synthesis of hydrophobic Ag_2_S

The synthesis of Ag_2_S was according to the reported method [33, 34] with slightly modified. 76.8 mg AgDDTC, 30 g ODE and 6 g DT were added into a 100 mL four-necked flask and vigorously stirred with passing through Ar, heated to 100 °C and kept for 5 min to remove the water then raised the temperature to 160 °C, after the solution color was completely dark, kept for 10 min and cooled down to room temperature, after centrifugal purification for 12,000 r/min by adding acetone, the resulting Ag_2_S was dissolved in chloroform and stored at 4 °C. Different concentrations of Ag_2_S were placed in centrifuge tubes and imaged by homemade NIR fluorescence imaging system.

The concentration of Ag in Ag_2_S was used to indicate the concentration of probe. A certain amount of Ag_2_S was taken out at room temperature, after the chloroform completely evaporated, HNO_3_ was added to dissolve it, titrated with NH_4_SCN (ferric ammonium alum as indicator), when the solution turned red, the titration end point was reached, then the concentration could be calculated.

### Synthesis of Ag_2_S@DSPE-PEG_2000_-FA (Ag_2_S@DP-FA)

According to the literature [35], 2 mL DMSO, 1 mL pyridine, 10 mg folic acid, 13 mg dicyclohexyl carbodiimide (DDC) and 4 mg DSPE-PEG_2000_-NH_2_ (DP-NH_2_) were joined in a 10 mL round bottom flask, stirred for 4 h in dark environment. The pyridine was removed by suspension, the remaining solution was dialyzed twice in 4.2 g/L NaHCO_3_ solution and three times in deionized water to remove free folic acid. Finally, the solution in dialysis bag was lyophilized to obtain DP-FA and stored in − 20 °C.

Ag_2_S@DP-FA was attached as positive probe. 4 mg lecithin, 6 mg polyoxyethylene stearate, 2 mg DP, 1 mg DP-FA and 700 μL Ag_2_S joined in a 10 mL round bottom flask, mixed at room temperature and slowly evaporated to dry, after that, added a small amount of PBS to dissolve. The method of synthesis phospholipid coating Ag_2_S negative probe without FA (Ag_2_S@DP) was consistent with the positive probe except adding DP-FA.

The probe’s stability was characterized by measuring the size and Zeta potential of Ag_2_S@DP-FA under 4, 25 and 37 °C respectively at 5, 10, 15, 20, 25 and 30 days.

### PAI and PTT evaluation of Ag_2_S@DP-FA

Different concentration Ag_2_S@DP-FA probes (0, 0.1, 0.2, 0.4, 0.8, and 1 mg/mL) embedded in 1% agar gel cylinders were performed quantitatively using single ELISA plate. The photoacoustic image (PAI) was measured at 744 nm and 0.6 mJ using a homemade PA system.

200 µL aqueous suspensions containing different concentrations of Ag_2_S@DP-FA probes were poured into different ELISA plates, and then illuminated by 808 nm laser with tunable output power density for 5 min. The increase of temperature was monitored and recorded by Thermal Camera.

### Cytotoxicity of the Ag_2_S@DP-FA

HeLa and A549 cells were homogenously inoculated in a 96-well plate for 8 groups, 5 repeated experiments for each group. Ag_2_S@DP-FA at concentrations of 0, 3.2, 6.4, 13, 25, 50, and 100 μg/mL were added to the cells. After incubating for 24 h, 20 μL MTT solution (5 mg/mL) was added to each well and kept for 4 h, the medium was sucked out and 150 μL DMSO was added to dissolved the violet crystals. The 96-well plate was shaken for 15 min, finally the absorbance was measured at 490 nm.

Long-term cytotoxicity of Ag_2_S@DP-FA was determined by clone formation assay [36]. According to the MTT method, the cells were seeded in a 96-well plate and incubated with different concentration Ag_2_S@DP-FA (0, 3.2, 6.5, 13, 25, and 50 mg/mL), besides, 4 parallels were set for each concentration. After 24 h incubation, about 2000 cells were inoculated in 6-well plates at each concentration. After 7 days culture, they were fixed with 2.5% glutaraldehyde for 30 min, washed the glutaraldehyde with PBS and added 5% crystal violet staining for 15 min. Finally, the number of cell communities was counted by Photoshop.

### In vitro FI and PAI

The positive HeLa cells and negative A549 cells were incubated with 50 µg/mL Ag_2_S@DP-FA and Ag_2_S@DP, respectively. After 12 h, the culture medium was aspirated and washed several times with PBS to ensure no free probe was presented. The cells were fixed with 4% paraformaldehyde and imaged by the homemade NIR fluorescence imaging system.

HeLa cells were divided into four groups, treated by Ag_2_S@DP-FA, Ag_2_S@DP, 1 mg FA for 1 h before Ag_2_S@DP-FA, and blank buffer respectively. After 2 h, the cells were washed by PBS for three times to clean up the uncombined probes. Then the cells were harvested by 0.25% trypsin–EDTA solution, and collected in 1% agar gel cylinders with single ELISA plates after centrifuging at 1000 rpm for 8 min and washing for three times with PBS. The PA signals were measured by the homemade PAI system.

### In vitro PTT

Positive and negative cells were seeded in a 24-well plate for 24 h prior to treatment respectively. 0.5 mL positive and negative probes dispersed in culture medium at the same Ag concentration of 25 μg/mL were added into the wells and incubated respectively. After 2 h, the cells were washed by PBS for three times to clean up the uncombined probes. Then the cells were irradiated for 5 min using 808 nm laser with a power density of 1.8 W/cm^2^. After that, the cells were stained by calcein acetoxymethyl ester (calcein-AM) for 5 min, and observed by inverted optical fluorescence microscope.

### TEM of cell uptake probes

Similar to fluorescence test, after incubation, HeLa and A549 cells were immobilized with 1% OsO_4_, then treated with different concentration ethanol (50, 70, 80, 85, 90, 95 and 100%), resined 48 h in the Epon812 at 60 °C. Ultra-thin slides were cut and observed by TEM.

### In vivo toxicity of the probe

50 BALB/c mice (male, SPF, 4 weeks) were divided into 5 groups randomly, half mice of each group were injected with Ag_2_S@DP probe (108 mg Ag/kg) by tail vein and the others were injected with the same volume of PBS. 300 μL blood was obtained at 0, 6 h, 1, 3, 7 and 15 days, 200 μL for liver enzyme analysis, AST and ALT, other 100 μL for blood analysis, RBC, WBC, PLT, and HGB. At the same time, the heart, liver, spleen, lung, kidney and small intestine were collected, fixed by 4% paraformaldehyde, dehydrated, embedded, sliced and HE staining, placed under the optical microscope for histopathological examination. All animal experiments were approved by the Animal Experimental Ethics Committee of Huazhong University of Science and Technology.

### In vivo targeting FI, PAI and PTT

Several BALB/c nude mice (male, SPF, 4 weeks) were randomly divided into 4 groups, group I and II were inoculated with positive HeLa cells, group III and IV were seeded with negative A549 cells. The dimensions of the tumors were monitored by vernier caliper every 2 days. Once the tumor volume grew to 80 mm^3^, Ag_2_S@DP-FA positive probe (108 mg Ag/kg) was injected into group I and III by tail vein, group II and IV were injected with the same amount of Ag_2_S@DP negative probe. The mice were imaged with the NIR fluorescence imaging system at 0 h, 5 min, 1, 2, 4, 12, 24, 36 and 48 h. At the same time point, the heart, liver, spleen, lung, kidney, small intestine and tumor of some mice in group I were collected and imaged by the homemade NIR fluorescence imaging system.

Twenty HeLa tumor-bearing nude mice were randomly divided into 4 groups for PTT. Group I was exposed to NIR laser with an output power density of 2.8 W/cm^2^ for 10 min after tail intravenously injection of Ag_2_S@DP-FA (108 mg Ag/kg); group II was irradiated for 10 min with injection of 600 μL saline solution; group III was tail intravenously injected with Ag_2_S@DP-FA (108 mg Ag/kg) without NIR laser irradiation; group IV was as a blank control without any treatment. Same as group I, positive probe was injected to another batch of tumor-bearing nude mice, in vivo photoacoustic imaging experiment was carried out at different time points. In the experiment, the nude mice were fixed on the stage of the photoacoustic system after anesthesia with 10 μL/g of urethane. The wavelength of laser was 744 or 532 nm and the laser energy per pulse was 200 nJ.

## Results and discussion

The NIR-II region emission of Ag_2_S quantum dot can greatly reduce the interference of spontaneous fluorescence from the organism, improve the signal to noise ratio. In addition, the absorption and scattering of light in the NIR-II region are less, so Ag_2_S has a stronger penetrating ability comparing with the traditional quantum dot, which can be used for the in vivo non-invasive imaging.

**Fig. 1:**
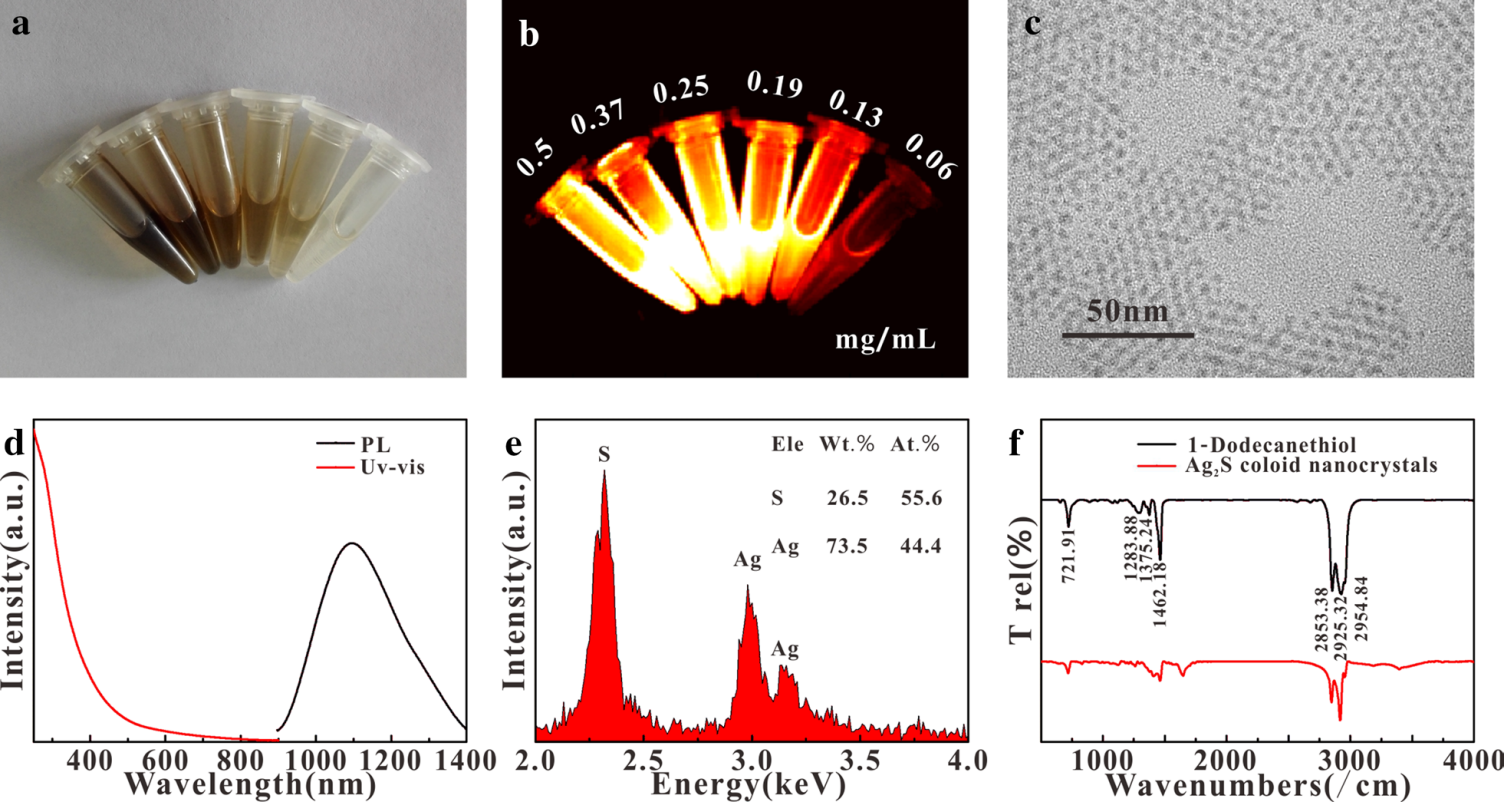
White light (**a**) and fluorescence (**b**) image, TEM (**c**), absorption and emission spectra (**d**), EDS (**e**) of Ag_2_S; the infrared spectrum of Ag_2_S and DT (**f**)

As the first step of this study, the hydrophobic Ag_2_S nanoparticle was synthesized (Fig. 1a), its fluorescence intensity increased significantly accompanying with increasing concentration (Fig. 1b). The spectral characterization showed a wide absorption and ~ 1100 nm emission (Fig. 1d). TEM showed that Ag_2_S had a relatively uniform size about 5 nm (Fig. 1c). EDS test showed that the sample consisted of Ag and S elements, but the molar ratio was less than 2:1 (Fig. 1e), this might be due to the fact that dodecanethiol (DT) contained S, increased the relative content of sulfur. The surface ligand on Ag_2_S was detected by infrared spectroscopy, results showed that Ag_2_S had a consistent characteristic of infrared spectroscopy with DT (Fig. 1f), indicating that DT had been successfully attached to the Ag_2_S surface.

**Fig. 2:**
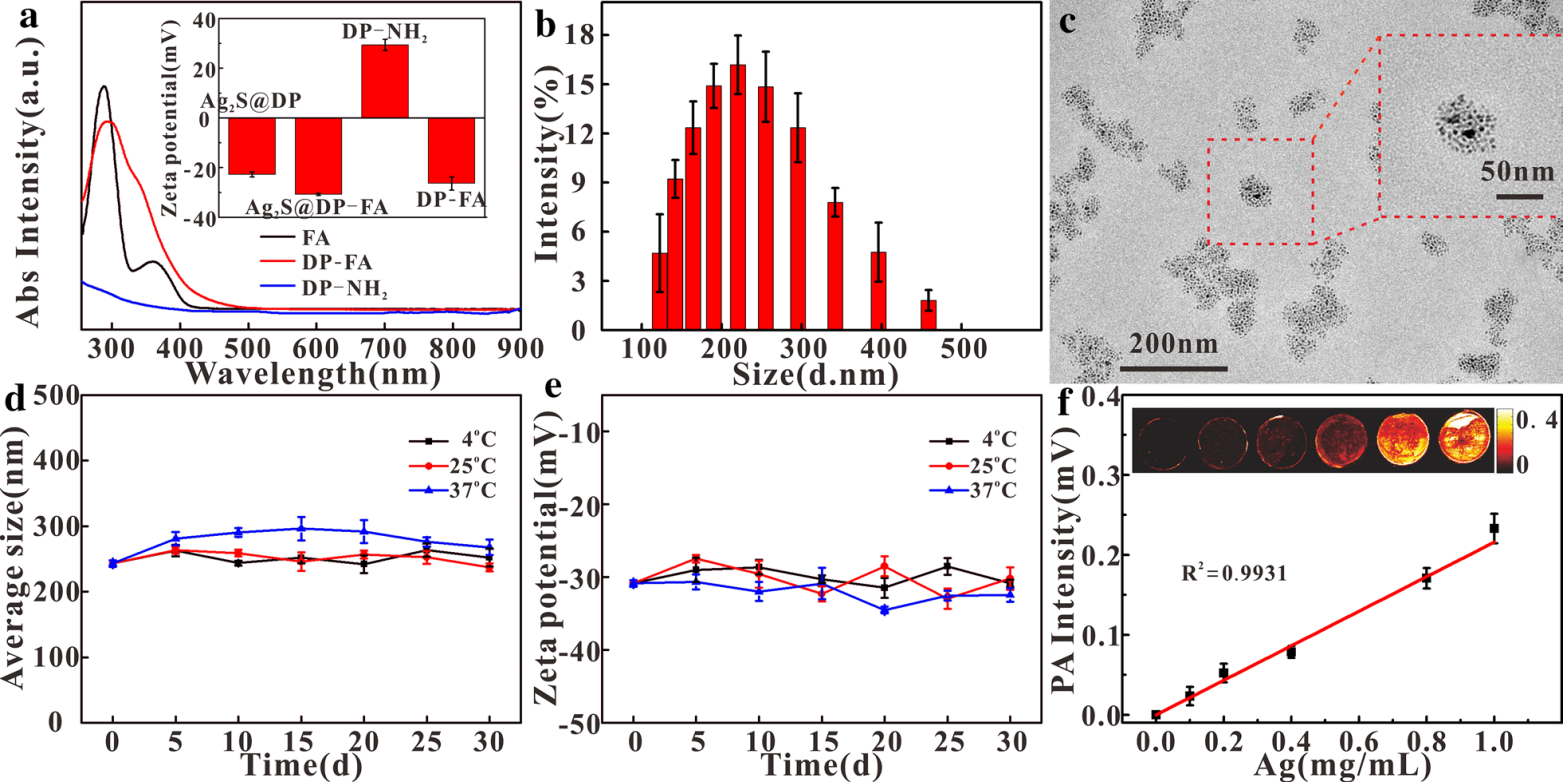
Absorption spectra of FA, DP-FA and DP-NH_2_ (**a**), insert: Zeta potentials of DP-NH_2_, DP-FA, Ag_2_S@DP-FA and Ag_2_S@DP (n = 5); dynamic light scattering results (**b**) (n = 5) and TEM (**c**) of Ag_2_S@DP-FA; the change of particle size (**d**) and Zeta potential (**e**) of Ag_2_S@DP-FA at different temperatures (n = 5); the PA response of the different concentrations of the probe (**f**) (n = 5), insert: PAI of probe

The target molecule FA was attached to DP-NH_2_ by DDC. To confirm whether or not FA had been successfully connected with DP-NH_2_, the absorption spectra of FA, DP-NH_2_, and DP-FA were measured (Fig. 2a). The results showed that the peak of FA was at 280 nm, while DP-FA had an obvious absorption at 280 nm comparing with the DP-NH_2_. In addition, the Zeta potential of these materials were also measured (Fig. 2a, insert). DP-NH_2_ was + 29.4 mV because it had amino group; for DP-FA, the FA possessed two carboxyl groups, even if one of them was linked with DP-NH_2_, the other carboxyl group also led to negative charge at − 26.4 mV. After modifying Ag_2_S particles, it was found that a higher negative potential (− 30.84 mV) was obtained than DP-modified particles without FA (− 21.8 mV). All these results confirmed that FA had been successfully conjugated with DP-NH_2_.

The size of Ag_2_S@DP-FA was about 200 nm measured by dynamic light scattering method (Fig. 2b). TEM showed a size of about 50 nm (Fig. 2c). This phenomenon might be due to the fact that the former was the hydrated size [37]. The stability of Ag_2_S@DP-FA was also measured (Fig. 2d, e). The results showed that the size and potential of Ag_2_S@DP-FA had no obvious change at different temperatures within 30 days, indicating a good stability.

Ag_2_S@DP-FA had strong absorption during the visible and NIR region. There were good PA response and PTT effect under certain wavelength laser irradiation. The in vitro photoacoustic and photothermal experiments of probe were carried out. Results showed it had obvious PA response under 744 nm laser irradiation. It could be seen that accompanying with the increasing Ag concentration, PA signal generated by Ag_2_S@DP-FA was strengthened (Fig. 2f) with a great linear relationship (R^2^ = 0.9931), indicating good ability of PAI.

**Fig. 3:**
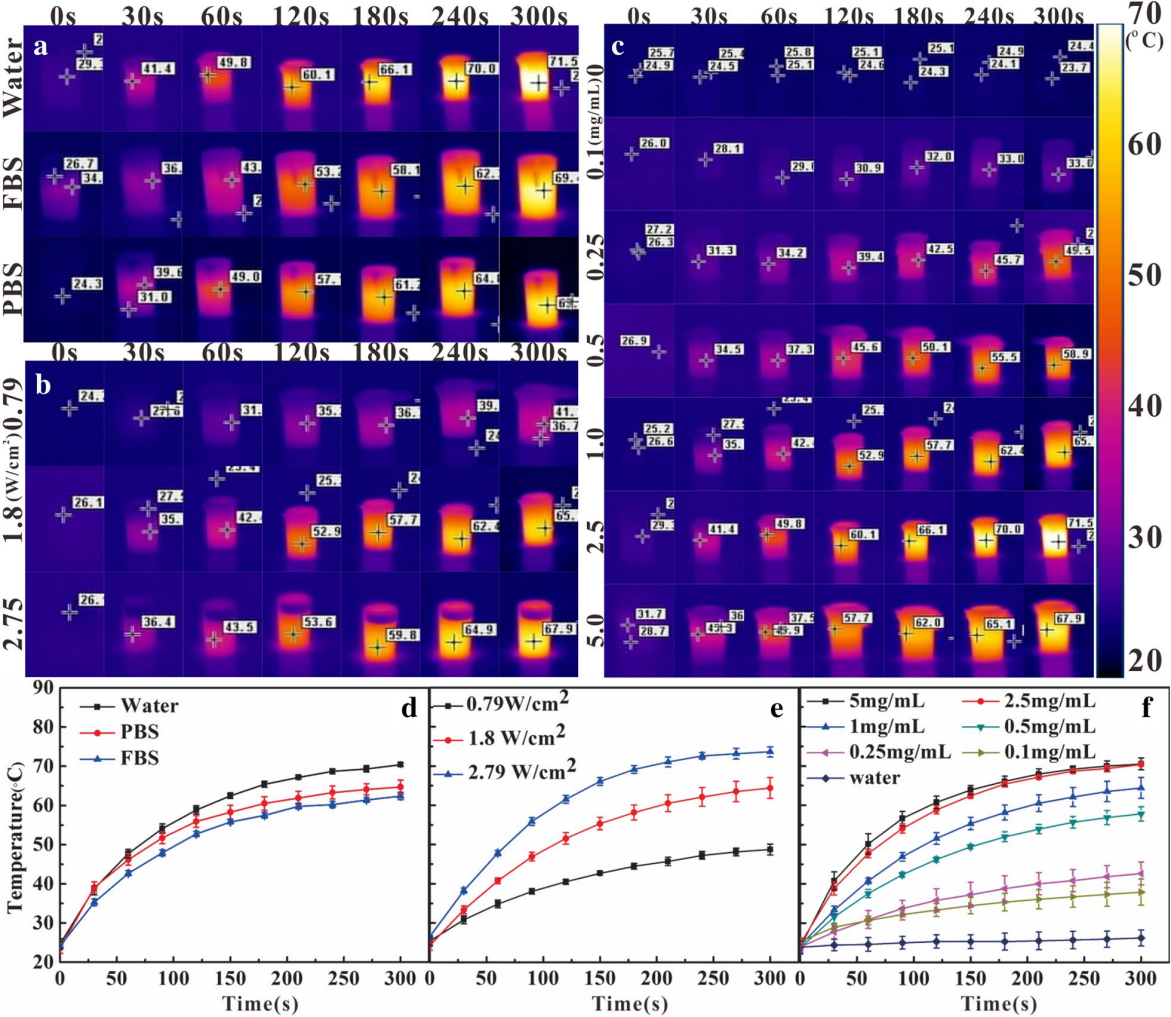
The picture of temperature evolution of 2.5 mg/mL Ag_2_S@DP-FA in different solvent under 1.8 W/cm^2^ laser over time (**a**); 1.0 mg/mL Ag_2_S@DP-FA in water under different laser power densities (**b**); different concentration Ag_2_S@DP-FA in water under 1.8 W/cm^2^ laser (**c**); **d**–**f** were the temperature evolution curves over time corresponding to (**a**, **b**, **c**), respectively (n = 5)

It was found that Ag_2_S@DP-FA (2.5 mg/mL) dispersed in deionized water, PBS and fetal bovine serum (FBS) all showed perfect photothermal conversion under NIR laser irradiation (Fig. 3a, d). And when the power density of NIR laser was increased, the temperature of aqueous dispersion of Ag_2_S@DP-FA (1.0 mg/mL) also increased (Fig. 3b, e). Furthermore, when Ag_2_S@DP-FA concentration increased, temperature of the aqueous dispersion could be elevated up to higher temperature (Fig. 3c, f). However, the temperature increment with the increase of concentration was not obvious when the concentration was above 2.5 mg/mL. Comparatively, the control experiment of deionized water showed the temperature increment less than 1 °C under the same experimental conditions. These results indicated that Ag_2_S probe had excellent photothermal conversion capability.

**Fig. 4:**
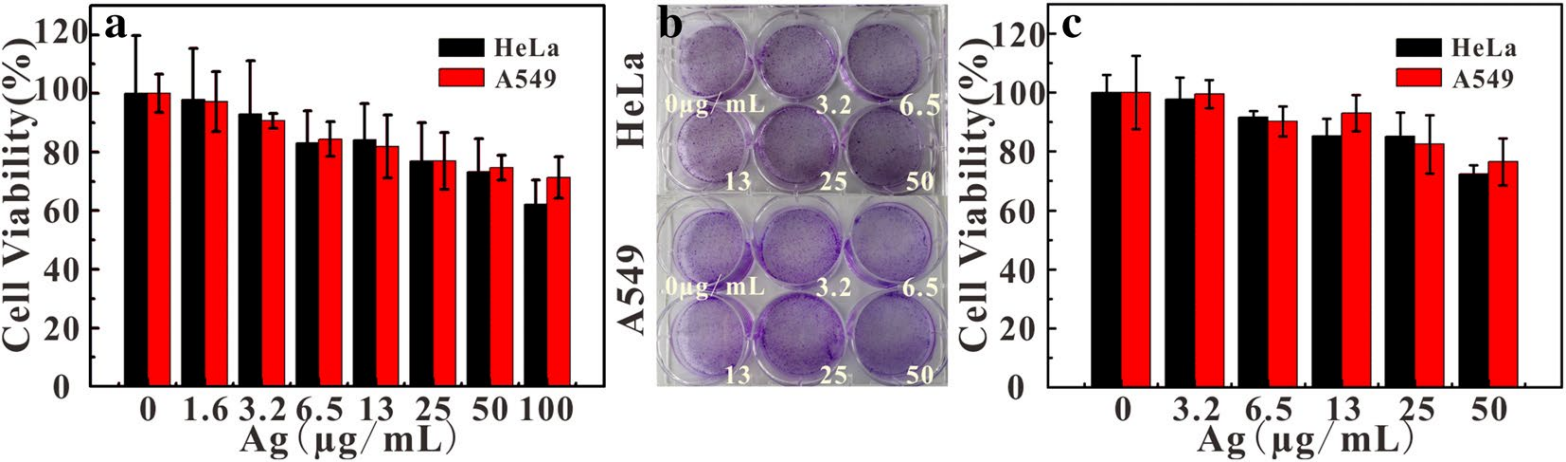
MTT test of Ag_2_S@DP-FA (**a**) (n = 5); white light results (**b**) and cell community data statistics (**c**) (n = 5) of colony formation assay method to detect the long-term toxicity of the probe

The toxicity of the probe was the key for application. The cytotoxicity of Ag_2_S@DP-FA probe was investigated by MTT assay. HeLa and A549 cells were incubated for 24 h with different concentration probe (Ag: 0, 1.6, 3.2, 6.5, 13, 25, 50, and 100 μg/mL). Succinate dehydrogenase in the mitochondria of living cells could reduce MTT to blue-violet crystals that could deposit in cells, whereas dead cells had no this function [38]. Results showed that when the concentration of Ag was up to 50 μg/mL, the cells still had near 80% survival (Fig. 4). Long-term cytotoxicity experiment was investigated by the colony formation assay. Cell viability was estimated through the number of cell population (Fig. 4b). It was found the results (Fig. 4c) were consistent with the MTT results, indicating that Ag_2_S @ DP-FA probe had a lower cytotoxicity.

**Fig. 5:**
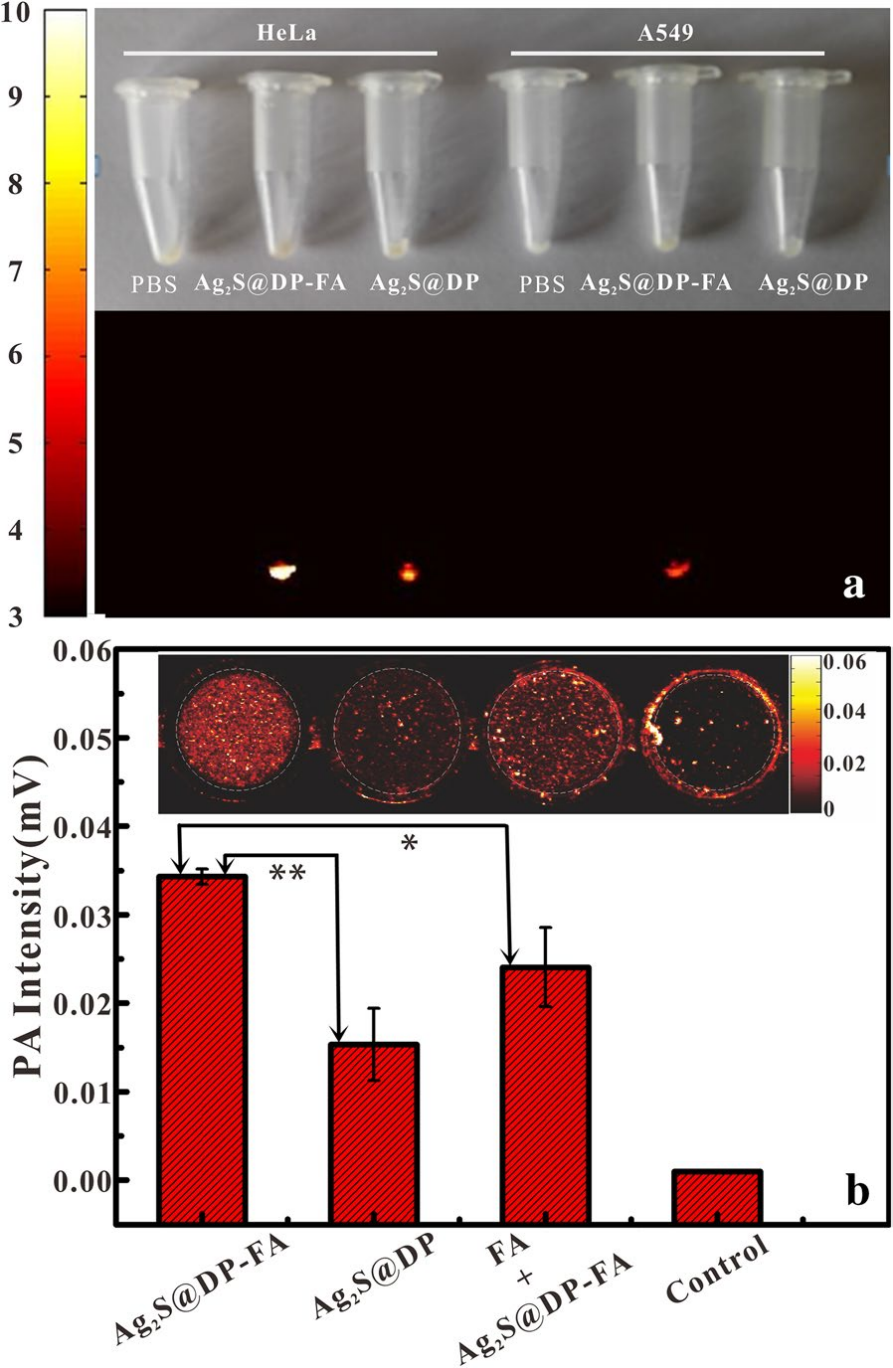
White light and FI results of different cells incubated with different probes (**a**) and photoacoustic response results of different probe-labeled positive HeLa cells (**b**) (n = 5), *meant significant difference (p < 0.05), **meant great significant difference (p < 0.01)

Then the active target ability of the probe was examined. HeLa and A549 cells were incubated with 50 μg/mL Ag_2_S@DP-FA and Ag_2_S@DP. The results showed that HeLa cells incubated with Ag_2_S@DP-FA were turned yellow and had the strongest fluorescence (Fig. 5a); while A549 cells incubated with Ag_2_S@DP-FA and HeLa cells incubated with Ag_2_S@DP were only observed faint yellow, and the fluorescence of the two was weak; the other groups looked white and no fluorescence was observed. The above results showed that Ag_2_S@DP-FA could specifically bind to FA receptors which high expressed on HeLa cells, and had no effective targeting ability for A549 cells which low expressed FA receptor. Likewise, negative probe Ag_2_S@DP had no effective targeting binding to HeLa and A549 cells. This experiment showed that the Ag_2_S@DP-FA probe could be used as an active targeting imaging diagnostic reagent for some tumors that high expressed FA receptors such as HeLa, and had the ability for target FI.

PAI was also conducted at the cellular level. HeLa cells were incubated with different probes for PAI. The results showed that both Ag_2_S@DP-FA and Ag_2_S@DP incubating HeLa cells had PA signals (Fig. 5b), indicating negative probe also combined to HeLa cells through nonspecific adsorption, this was consistent with the FI results (Fig. 5a). But there was great significant difference between them (p < 0.01), meant Ag_2_S@DP-FA combined to HeLa cells more easily than Ag_2_S@DP. To verify this further, FA was added into the cell culture flask before 1 h of adding Ag_2_S@DP-FA, significant difference was obtained (p < 0.05). It was because that the FA receptors on the cell surface were combined with free FA firstly, resulting in subsequent Ag_2_S@DP-FA could not combine to HeLa. These experiments above confirmed that Ag_2_S@DP-FA had good active target ability.

**Fig. 6:**
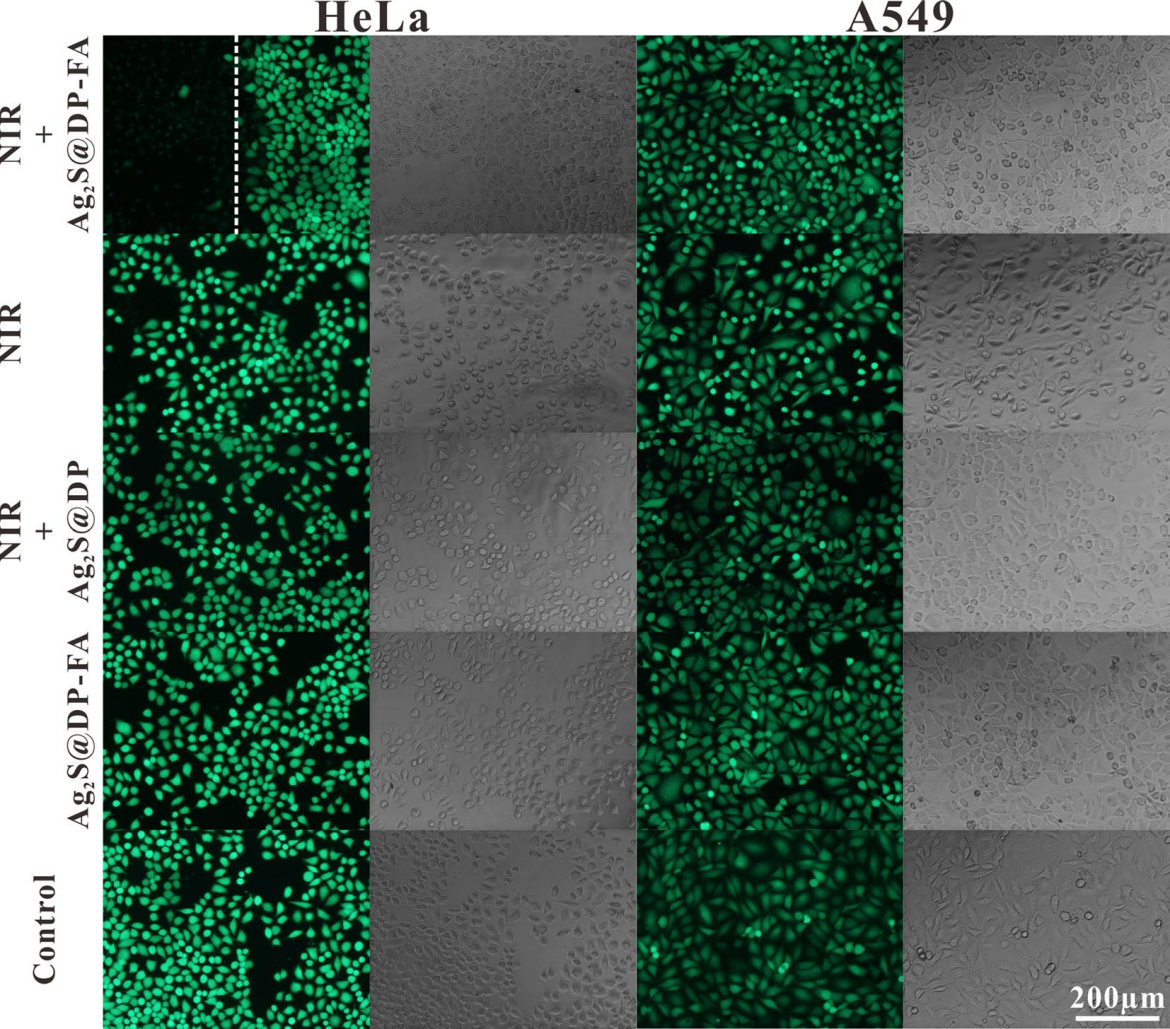
HeLa and A549 were incubated with positive probe (Ag_2_S@DP-FA) or negative probe (Ag_2_S@DP), and then treated with or without NIR laser irradiation. Cells were stained by calcein-AM. Laser power was 1.8 W/cm^2^, and irradiation time was 5 min. White dotted line was the border of laser irradiation

FA as a target molecule on the probe, its targeting could be also verified by the effect of PTT. When Ag_2_S@DP-FA positive probe was incubated with HeLa cells and irradiated with laser for 5 min, calcein staining revealed that the cells in the non-irradiated region had significant fluorescence, while the irradiated area showed only extremely weak fluorescence (Fig. 6). Calcein is a cell dyeing reagent which could stain live cell producing green emission except dead one [39]. If HeLa cells were only incubated with Ag_2_S@DP-FA without any irradiation, the cells showed obvious green fluorescence. To rule out the possibility of death by laser, HeLa cells were exposed to laser irradiation directly under same factors, the results showed that the cells still had bright green fluorescence, indicating that cell death was caused by the thermal effect of Ag_2_S@DP-FA. In order to investigate the ability of active targeting of probe, HeLa cells were incubated with Ag_2_S@DP, the results showed after laser irradiation the cells still had green fluorescent, meant negative probe could not target HeLa effectively. It seemed opposite to the results of PAI (Fig. 5b), but it was understandable, although there was nonspecific adsorption of Ag_2_S@DP, not in sufficient quantities to enhance temperature to kill cells, so they were still alive. For control experiments, no matter what kind of conditions above, as low FA receptor expression cell, A549 all present obvious green fluorescence. These experiments proved that only HeLa cells incubated with Ag_2_S@DP-FA could be killed by laser irradiation, which could fully demonstrate Ag_2_S@DP-FA had good ability of active targeting and PTT.

**Fig. 7:**
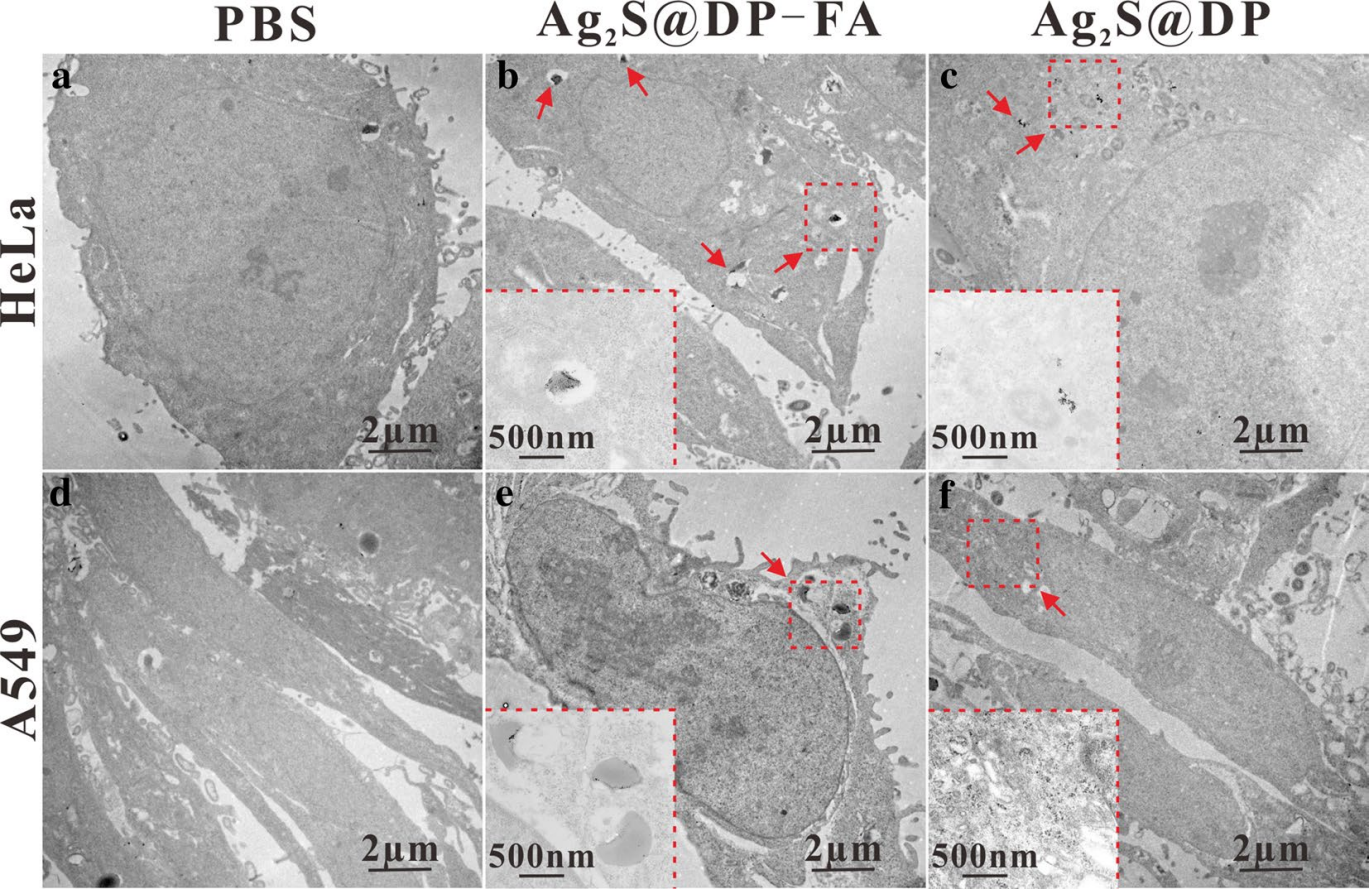
TEM of cells incubated with probes. **a** HeLa + PBS; **b** HeLa + Ag_2_S@DP-FA; **c** HeLa + Ag_2_S@DP; **d** A549 + PBS; **e** A549 + Ag_2_S@DP-FA; **f** A549 + Ag_2_S@DP. Inserts in **b**, **c**, **e** and **f** were the corresponding local magnification

TEM was used to further investigate the uptake of different probes by HeLa cells and A549 cells (Fig. 7). The results showed that comparing with the PBS control group (Fig. 7a, d), black particles appeared in the cytoplasm of HeLa (Fig. 7b) and A549 (Fig. 7e) cells in Ag_2_S@DP-FA group, which confirmed that both HeLa and A549 cells could ingest a certain amount of Ag_2_S@DP-FA probes by endocytosis, the amount of probes in HeLa cells was significantly higher than A549 cells. This result was consistent with the results of the FI and PAI because HeLa cells expressed more FA receptors than A549 cells, and thus could bind more targeting probes. There were also a few probes in the HeLa and A549 cytoplasm in Ag_2_S@DP group, and probes in the HeLa cells (Fig. 7c) were slightly more than A549 cells (Fig. 7f). This was also consistent with the results of FI. Overall, the amount of Ag_2_S@DP-FA probes in the HeLa cytoplasm was significantly the highest than the other control groups, this was consistent with the FI and PAT and PTT experiments, indicating that the Ag_2_S@DP-FA probe had a good active targeting ability and could be used as a target imaging diagnostic reagent.

**Fig. 8:**
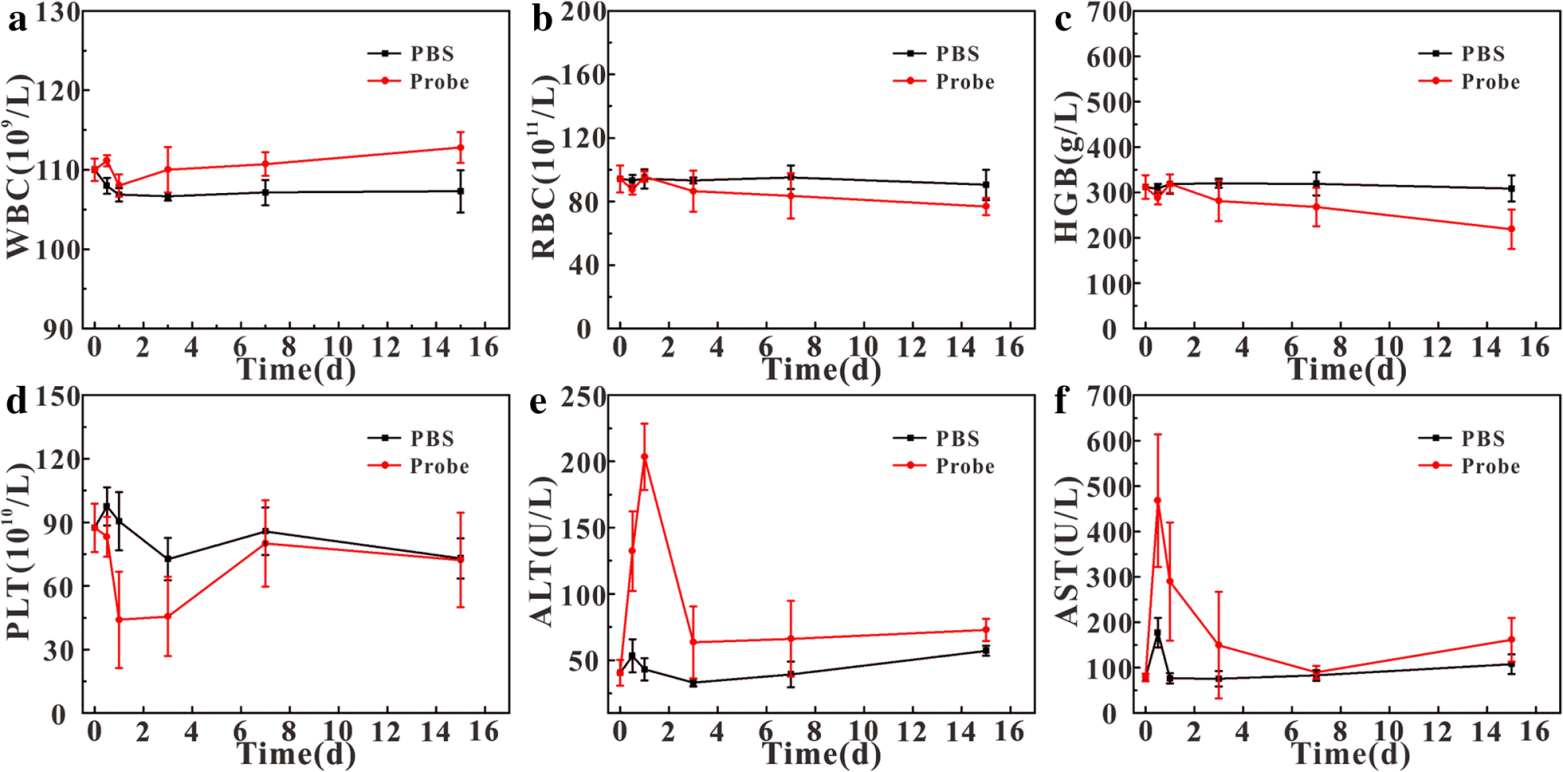
Blood sample analysis after injection (n = 5). **a** WBC; **b** RBC; **c** HGB; **d** PLT; **e** ALT; **f** AST

In order to evaluate the in vivo safety of the probe, the blood biochemical indexes in mice were examined after injection of the probe. The results showed that there was no significant difference in white blood cell (WBC) (Fig. 8a), red blood cell (RBC) (Fig. 8b), and hemoglobin (HGB) (Fig. 8c) between the probe group and the PBS control group. The platelet (PLT) in probe group (Fig. 8d) was significantly reduced after 1 day but returned to normal at 7 days, indicating the probe did not cause irreversible damage to the mice. In addition, the effect of probe on liver function was investigated by testing the level of alanine aminotransferase (ALT) (Fig. 8e) and aspartate aminotransferase (AST) (Fig. 8f). The results showed that ALT and AST contents significantly changed after injection then backed to normal levels at 3 days, PBS group also had similar change. The reason for this short rise was due to the use of anesthetics, which might increase liver enzyme activity and cause AST and ALT in the mice blood to be temporarily rising for several hours [40]. The above results showed that our synthetic probe had good biosafety.

**Fig. 9:**
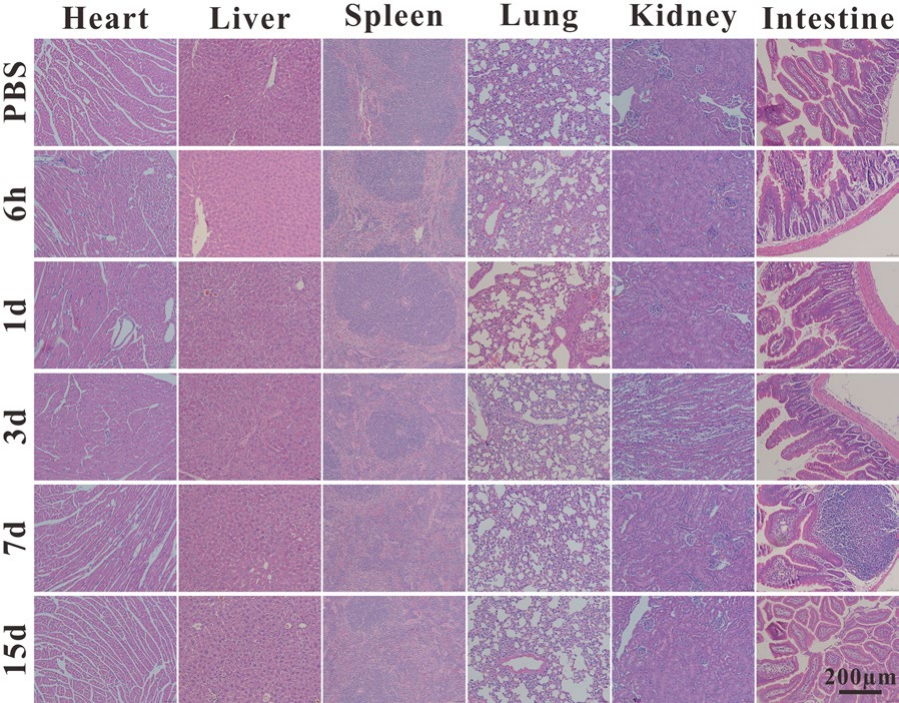
The HE staining results of the main organ after injecting PBS and probe for 6 h, 1, 3, 7, 15 days shown in from top to bottom, from the left to right was heart, liver, spleen, lung, kidney, and intestine

In addition to blood analysis, the effects of probes on mice organs were also examined. Comparing the HE staining results of PBS group with probe group at 6 h, 1, 3, 7 and 15 days after injection (Fig. 9), it was found that the tissue structure of heart, liver, spleen, lung, kidney, and small intestine did not change significantly, indicating that the probe had little effect on these organs, further proved that our probe had good biosecurity.

**Fig. 10:**
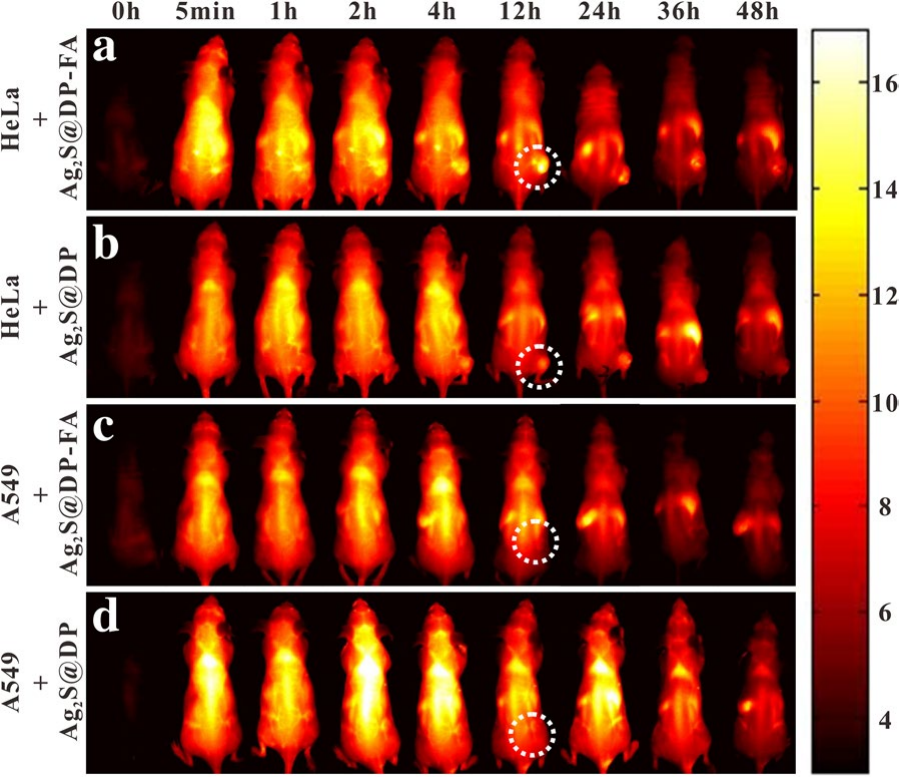
FI of the target tumor in the nude mice at 0 h, 5 min, 1, 2, 4, 8, 12, 24, 36 and 48 h (the tumor site in the dashed line cycle). **a** HeLa tumor-bearing nude mice + Ag_2_S@DP-FA; **b** HeLa tumor-bearing nude mice + Ag_2_S@DP; **c** A549 tumor-bearing nude mice + Ag_2_S@DP-FA; **d** A549 tumor-bearing nude mice + Ag_2_S@DP

After that, the in vivo active targeting imaging ability of the probe was examined. The Ag_2_S@DP-FA and Ag_2_S@DP probes were injected into HeLa tumor-bearing nude mice and A549 tumor-bearing nude mice by tail vein, and they were imaged by homemade NIR fluorescence imaging system at different times. The results showed that Ag_2_S@DP-FA spread all over HeLa tumor-bearing nude mouse after 5 min, the tumor site had obvious fluorescence after 1 h and showed brightest fluorescence at 12 h, then it began to decay, after 36 h, the tumor site still had a certain intensity of fluorescence (Fig. 10a). While the HeLa tumor-bearing nude mouse injected with Ag_2_S@DP had a weaker fluorescence in tumor at 4 h, and the fluorescence disappeared at 24 h (Fig. 10b). As for A549 tumor-bearing nude mice, no significant fluorescence enhancement were observed at the tumor sites (Fig. 10c, d), indicating that the probe did not enrich at the tumor sites. This series of in vivo fluorescence imaging were consistent with in vitro FI results, further showed that Ag_2_S@DP-FA probe had a good active targeting imaging capability.

**Fig. 11:**
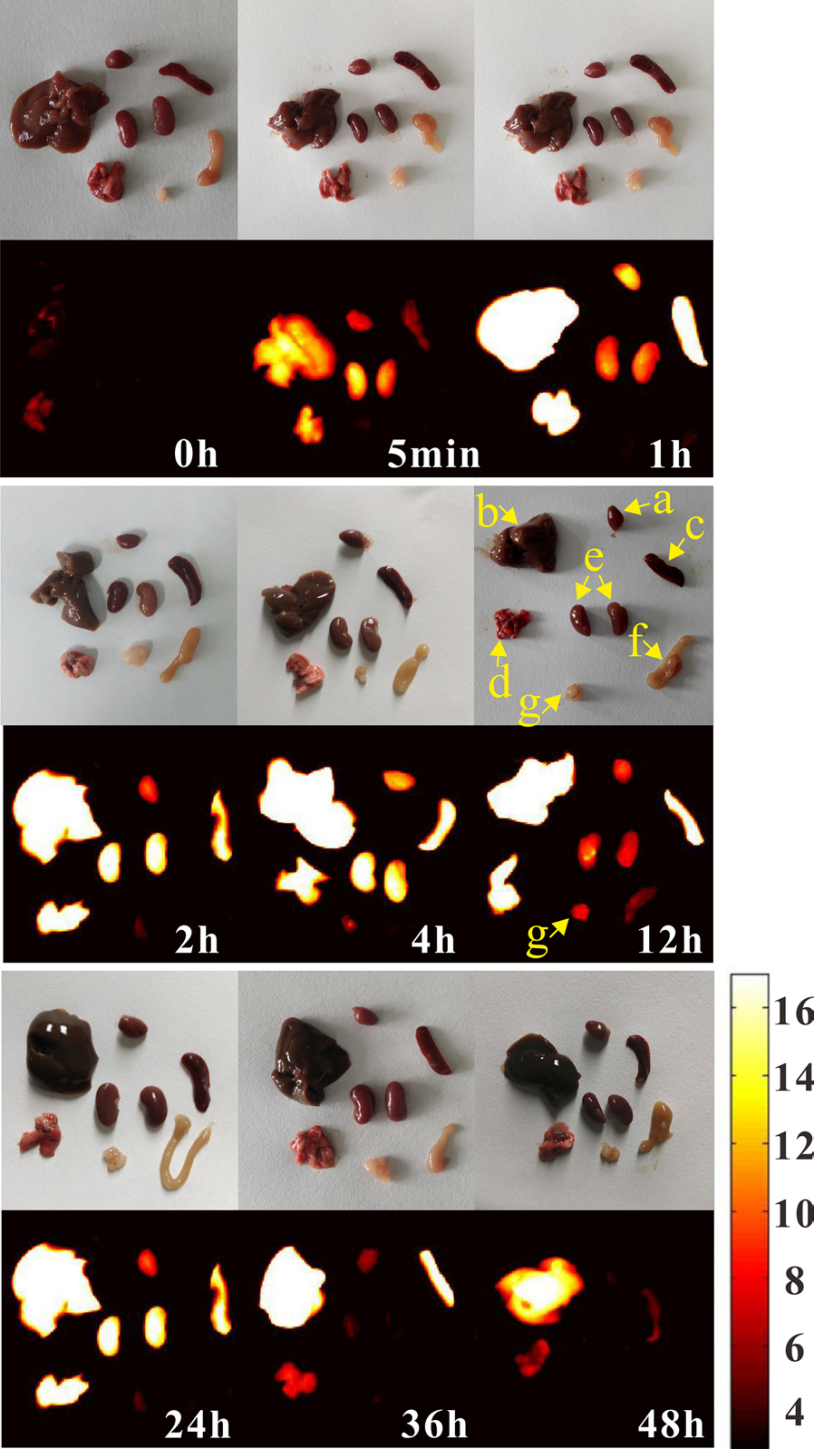
White and fluorescence imaging of heart (a), liver (b), spleen (c), lung (d), kidney (e), small intestine (f), and tumor (g) in HeLa tumor-bearing nude mice at 0 h, 5 min, 1, 2, 4, 8, 12, 24, 36 and 48 h after injection of Ag_2_S@DP-FA

Next, the distribution of the probe in the nude mice was examined. At different times, the main organs and tumor were collected for FI. The results showed that the probe had more residues in the liver, spleen and lungs, and had fewer residues in the heart, kidneys and small intestine (Fig. 11). With the extension of time, the probe gradually discharged from the organs. Tumor site began to light at 1 h, the fluorescence effect was more obvious at 4 h, and reached the maximum at 12 h, then began to weaken and continued to 36 h, same with in vivo targeted FI.

**Fig. 12:**
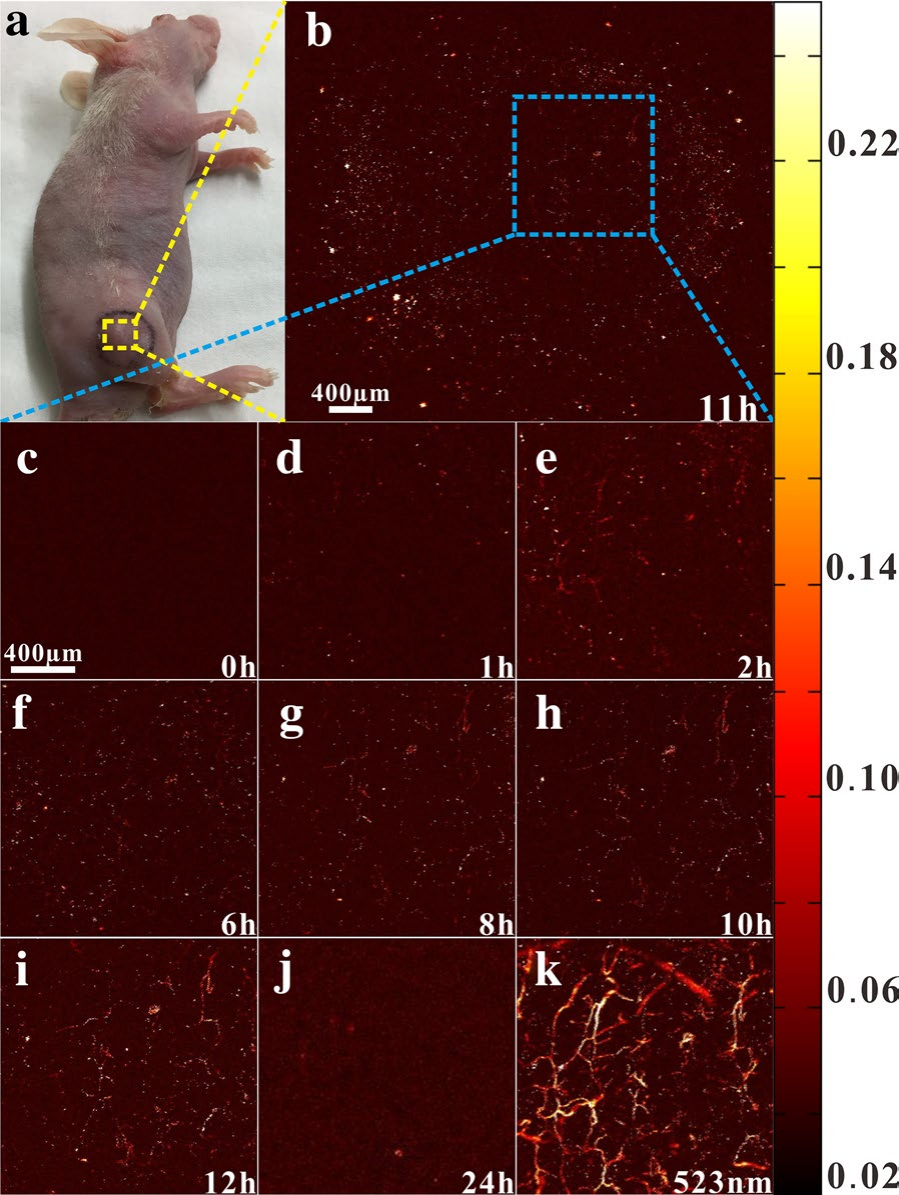
In vivo PAI of the nude mouse tail intravenously injected with Ag_2_S@DP-FA (**a**); the whole tumor after injecting 11 h under 744 nm laser excitation (**b**); the same part of tumor at different times under 744 nm laser excitation (**c**–**j**); the same part of tumor under 523 nm laser excitation (**k**)

Once the tumor volumes were about 80 mm^3^, in vivo PAI of nude mice tail intravenously injected with Ag_2_S@DP-FA was carried out using 744 nm laser. The PAI system has high lateral resolution (~ 4 µm), it showed before tail intravenous injection, there was no PA signal on the tumor (Fig. 12c). After injection, PA signal intensity of tumor showed significant enhancement comparing with the background (Fig. 12b). It indicated that the accumulation of Ag_2_S@DP-FA into the tumor was increased in the initial several hours, and reached the highest after 12 h, the PA response became weak after 24 h (Fig. 12d–j). This result was consistent with the FI. To determine whether or not the imaging was indeed at a vascular site of the tumor, PA signal of the same tumor region was also detected under 523 nm, it showed that there were rich blood vessels due to strong PA response of hemoglobin at this wavelength (Fig. 12k), the pattern was consistent with the PAI at 12 h (Fig. 12i). It demonstrated PA signal at 744 nm was derived from Ag_2_S@DP-FA targeted to the tumor.

**Fig. 13:**
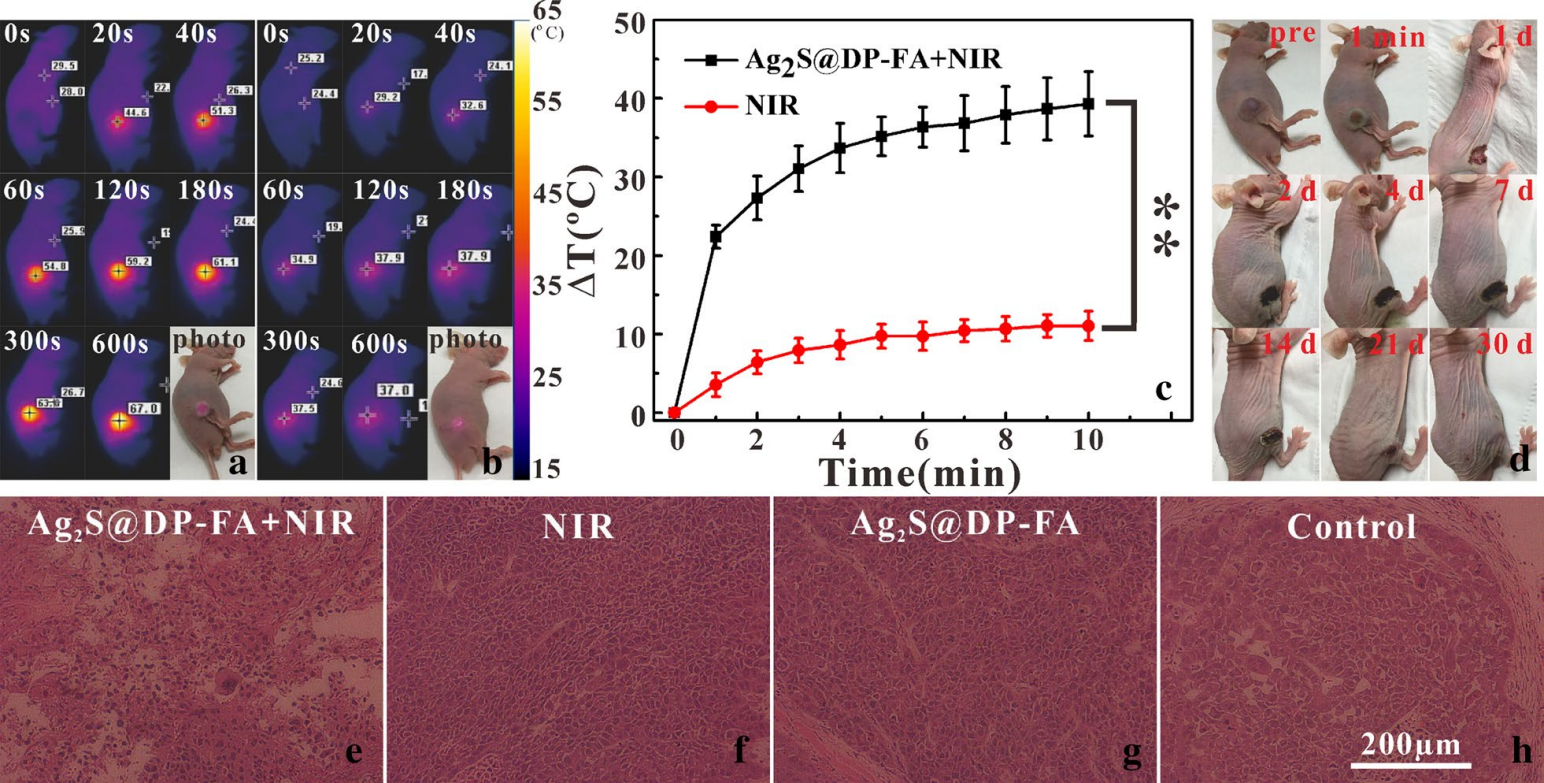
Infrared thermal images of HeLa tumor-bearing nude mouse tail intravenously injected with Ag_2_S@DP-FA (**a**) or saline (**b**) on different times under 808 nm laser irradiation; the temperature of tumor evolution curves over time (**c**) (n = 5), **meant great significant difference (p < 0.01); representative photos of tumor-bearing nude mouse after treatments over time (**d**); H&E stain of tumor tissues from different treatment groups (**e**–**h**)

Based on efficient in vitro PTT effect and in vivo FI and PAI, in vivo PTT was carried out further. After undergoing laser irradiation for 10 min, the temperature of bearing tumor on nude mouse tail intravenously injected with Ag_2_S@DP-FA could rise up to 67 °C (Fig. 13a, c), which would kill the tumor adequately. As control group, the temperature of bearing tumor on nude mouse tail intravenously injected with saline just rose up 10 °C (Fig. 13b, c), there was great significant difference between them (p < 0.01), indicating that the Ag_2_S@DP-FA probe targeted to the tumor exerted significant PTT effect. The region of tumor treated with Ag_2_S@DP-FA was charred immediately upon laser irradiation and further became black scar after 1 day, as time went on, the scar of tumor was healed little by little, and became remarkable recovery finally (Fig. 13d), suggesting the cancer was killed by high temperature. Actually, the group injected with Ag_2_S@DP-FA and underwent laser irradiation was completely cured without reoccurrence after 60 days investigation. H&E stain of tumor tissue was further conducted to reveal the therapeutic mechanism. It was found that apparent extensive necrosis appeared in the group treated with Ag_2_S@DP-FA and laser irradiation, however, no obvious malignant necrosis was found in the other three groups (Fig. 13e–h). All the results demonstrated that Ag_2_S@DP-FA had good ability of target and PTT.

**Fig. 14:**
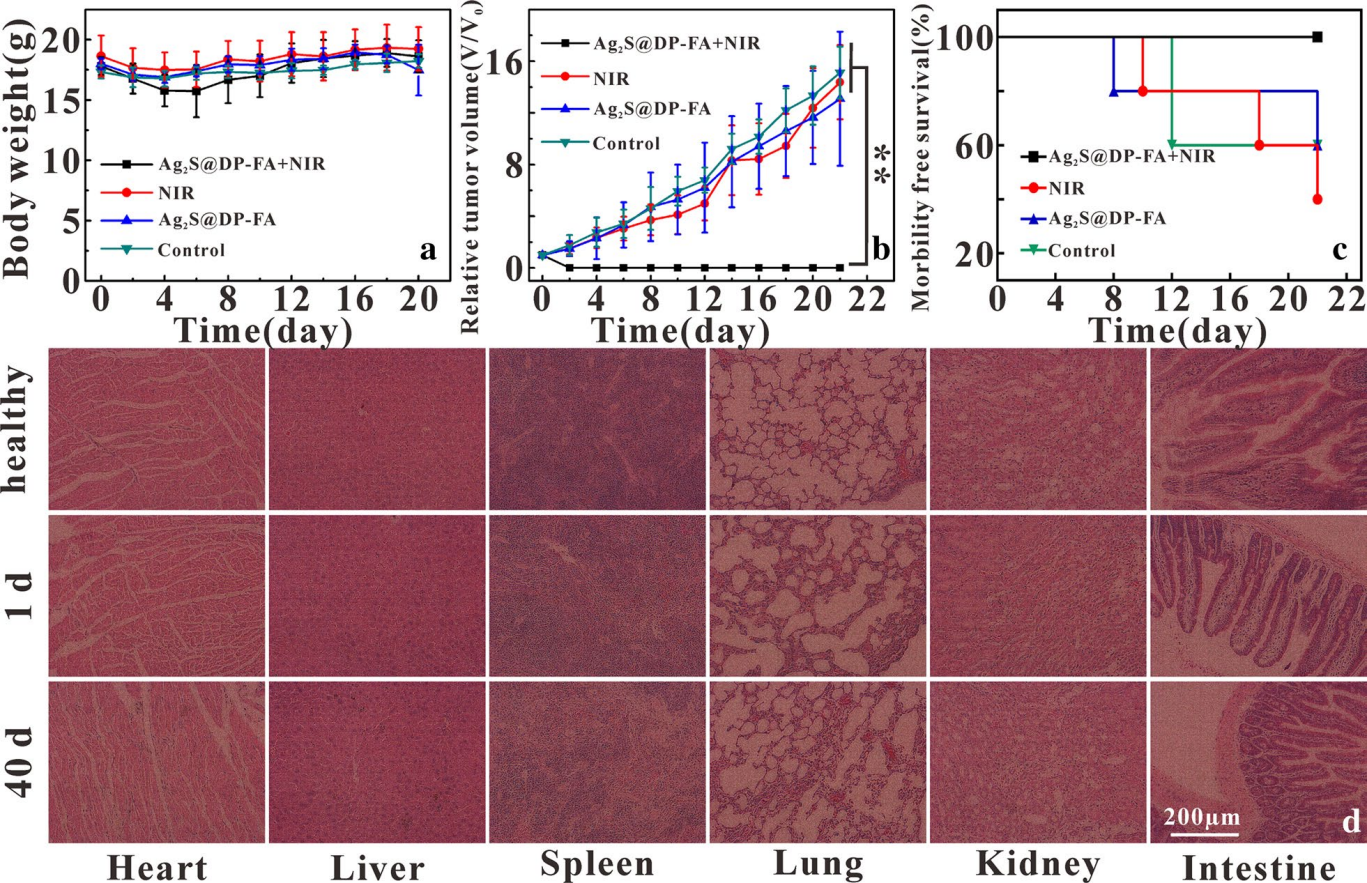
Body weight curves (**a**), tumor growth curves (**b**) and survival curve (**c**) after different treatments (n = 5), **meant great significant difference (p < 0.01); H&E stained images of major organs from healthy nude mice, 1 and 40 days after Ag_2_S@DP-FA and laser irradiation treatment mice, respectively (**d**)

The in vivo treatment toxicity was always a great concern for nanomaterials used in biomedicine, a series of works on it had been done. The changes in body weight of nude mice in four different treatment groups were recorded, and the results showed that PTT did not significantly alter body weight (Fig. 14a), indicating that treatment was safe, no significant side effect on the growth of nude mice. The tumor volumes were also measured (Fig. 14b). All irradiated tumors on mice injected with Ag_2_S@DP-FA disappeared and were cured without recurrence, there was great significant difference between the experiment group and the control groups (p < 0.01). In marked contrast, the other three groups all showed similarly rapid tumor growth. Considering a survival cutoff criteria, when the aggregate tumour burden > 1 cm in diameter, the mouse was euthanasia. The survival curve showed that mice in the three control groups died successively after 8 days, while mice in the treated group were survived over 22 days without a single death (Fig. 14c). Major organs of Ag_2_S@DP-FA treated mice whose tumors were eliminated by the PTT were collected after 1 and 40 days for histology analysis. No noticeable signal of organ damage and tumor spread were observed from H&E stained organ slices (Fig. 14d) comparing with healthy nude mice. These works all meant that our therapy was effective without obvious toxicity.

## Conclusion

In this paper, aqueous probe Ag_2_S@DP-FA with good dispersibility and stability was prepared by coating hydrophobic Ag_2_S with the mixture of FA modified DP and the other polymers. In vitro and in vivo fluorescence, photoacoustic imaging and photothermal therapy have demonstrated that Ag_2_S@DP-FA was a safe, integrated diagnosis and treatment probe with multi-mode imaging, photothermal therapy and active targeting ability, which had a great application prospect in the early diagnosis and treatment of tumor.

## Additional files

**Additional file 1** Video of in vivo PAI of the nude mouse before tail intravenously injection of Ag_2_S@DP-FA under 744 nm laser excitation.

**Additional file 2** Video of in vivo PAI of same part of tumor on the nude mouse after injection for 12 h under 744 nm laser excitation.

**Additional file 3** Video of in vivo PAI of same part of tumor on the nude mouse under 523 nm laser excitation.

## Abbreviations

Ag_2_S@DP-FA: Ag_2_S@DSPE-PEG_2000_-FA
FA: folic acid
DP: DSPE-PEG_2000_
CT: computed tomography
PAI: photoacoustic imaging
MRI: magnetic resonance imaging
PET: positron emission computed tomography
FI: fluorescence imaging
NIR: near-infrared
PA: photoacoustic
PTT: photothermal therapy
DT: dodecanethiol
FBS: fetal bovine serum
WBC: white blood cell
RBC: red blood cell
HGB: hemoglobin
PLT: platelet
ALT: alanine aminotransferase
AST: aspartate aminotransferase
DMSO: dimethyl sulfoxide
ODE: octadecene
DDC: dicyclohexyl carbodiimide
calcein-AM: calcein acetoxymethyl ester
